# A Bi-Invariant Statistical Model Parametrized by Mean and Covariance on Rigid Motions

**DOI:** 10.3390/e22040432

**Published:** 2020-04-10

**Authors:** Emmanuel Chevallier, Nicolas Guigui

**Affiliations:** 1 Institut Fresnel, Aix-Marseille University, CNRS, Centrale Marseille, 13013 Marseille, France; 2 Université Côte d’Azur, Inria Epione, 06902 Sophia Antipolis, France; nicolas.guigui@inria.fr

**Keywords:** wrapped distributions, rigid motions, Euclidean groups, differential of the exponential, moment-matching estimator, density estimation, sampling

## Abstract

This paper aims to describe a statistical model of wrapped densities for bi-invariant statistics on the group of rigid motions of a Euclidean space. Probability distributions on the group are constructed from distributions on tangent spaces and pushed to the group by the exponential map. We provide an expression of the Jacobian determinant of the exponential map of SE(n) which enables the obtaining of explicit expressions of the densities on the group. Besides having explicit expressions, the strengths of this statistical model are that densities are parametrized by their moments and are easy to sample from. Unfortunately, we are not able to provide convergence rates for density estimation. We provide instead a numerical comparison between the moment-matching estimators on SE(2) and $R^{3}$, which shows similar behaviors.

## 1. Introduction

This work is an extended version of the conference paper [1], focused on SE(2). We provide here a formula for SE(n) with arbitrary n≥2, and a numerical evaluation of the convergence of the moment-matching density estimator on SE(2).

Probability density estimation problems generally fall in one of two categories: estimating a density on a Euclidean vector space or estimating a density on a non-Euclidean manifold. In turn, estimation problems on non-Euclidean manifolds can be divided in different categories depending on the nature of the manifold. The two main classes of non-Euclidean manifold encountered in statistics are Riemannian manifolds and Lie groups. On Riemannian manifolds, the objects studied in statistics should be consistent with the Riemannian distance. For instance, means of distributions are defined as points minimizing the average square Riemannian distances. On a Lie group, the objects should be consistent with the group law. Direct products of compact Lie groups and vector spaces for examples belong to both categories, they admit a Riemannian metric invariant by left and right multiplications. However, in full generality, Lie groups do not admit such nice metrics, hence the need for statistical tools based solely on the group law and not on the Riemannian distance.

The definition of a statistical mean on Lie groups was addressed by Pennec and Arsigny in [2] where authors define bi-invariant means on arbitrary Lie groups as exponential barycenters [3]. Once the bi-invariant mean is defined, higher order bi-invariant centered moments can be defined in the tangent space at the mean. We build on this notion of moments to address the problem of constructing statistical models on SE(n), the group of direct isometries of $R^{n}$. The wrapped distributions model we propose has several advantages. First, it is stable under left and right multiplications. Second, densities have explicit expressions and are parameterized by their mean and covariance rather than their concentration matrix as for normal distributions defined in [4]. Third, the densities are easy to sample from. To do so, we construct wrapped densities on SE(n) similar to the densities defined in [5,6,7,8,9,10,11] on Riemannian manifolds. Similar types of probability distributions have already been considered for robotics applications on SE(3) to model uncertainty in motion estimation, see for instance [12].

Harmonic analysis is another well-known approach to density estimation, see [13] for SE(2) and [14,15,16] on other manifolds. Beside the technicalities and numerical difficulties introduced by harmonic analysis on non-abelian and non-compact groups, the main motivation for using wrapped distributions over harmonic analysis techniques, is that it enables the definition of parametric models.

This work is based on two facts. First, the exponential map can be translated from the identity element to any point of the group regardless of the choice of left or right multiplication. This property was already of primary importance in the construction of the bi-invariant mean [2] and enables the definition of bi-invariant estimation procedures. The second important fact is that the Jacobian of the exponential map on SE(n) admits a closed form expression which we compute in Section 3.2. This Jacobian provides an easy way to define probability densities with explicit expressions on the group by pushing densities from tangent spaces using the exponential map.

Unfortunately, the literature on convergence of bi-invariant moments on Lie group is still very limited. Therefore, we were not able to characterize the convergence of estimators using the proposed model. Instead, we compared numerically the convergence of the moment-matching estimator on SE(2) and on $R^{3}$.

The paper is organized as follows. Section 2 describes the group of direct isometries of the Euclidean space. Section 3 includes relevant properties of the exponential mapping and the computation of the Jacobian determinant. Section 4 recalls the definitions of the first and second centered moments on a Lie group. A statistical model together with a sampling and an estimation procedure is introduced in Section 5. Section 6 concludes the paper.

## 2. Euclidean Groups

For a condensed introduction to Lie group theory for robotics, see [17], and for several relevant calculations on low-dimensional rigid motions, see the series of notes [18,19,20].

SE(n) is the set of all direct isometries of the Euclidean space $R^{n}$. The composition law of maps makes SE(n) a group. For each element *g* of SE(n) there are a unique rotation *R* and a unique vector *t* such that

g(u)=Ru+t,

hence the isometry *g* can be represented by the couple (R,t). The group structure of SE(n) is not a direct product between the special orthogonal group and the group of translations, but a semi direct product with translations as the normal subgroup:

$$SE\left(n\right)=SO\left(n\right){⋉}_{\phi }R^{n}$$

$$\left(R,t\right)\left(R^{\prime },t^{\prime }\right)=\left(RR^{\prime },\phi_{R}\left(t^{\prime }\right)+t\right)$$

where we simply have $\phi_{R}=R$. Let $\Psi_{\left(R,t\right)}$ denote the conjugation by (R,t). A short calculation gives

$$\Psi_{\left(R,t\right)}\left(R^{\prime },t^{\prime }\right)=\left(R,t\right)\left(R^{\prime },t^{\prime }\right){\left(R,t\right)}^{-1}=\left(RR^{\prime }R^{t},-RR^{\prime }R^{t}t+Rt^{\prime }+t\right).$$

Recall that $Ad_{R,t}=d{\left(\Psi_{\left(R,t\right)}\right)}_{e}.$ Hence, after unfolding the elements of the Lie algebra se(n) into column vectors, the matrix representation of $Ad_{R,t}$ is given by

(1) $$Ad_{\left(R,t\right)}:\left(\begin{array}{cc} Ad_{R} & 0\\ C & R \end{array}\right)$$

where *C* is a *n* by $\frac{n(n-1)}{2}$ matrix, $Ad_{R}$ is the adjoint representation of rotations. The structure of this adjoint matrix implies first that SE(n) is unimodular, i.e., admits a bi-invariant measure and the derivative of the exponential admits an explicit expression as we will see in Section 3.2. To see that SE(n) is unimodular, consider a left-invariant volume form ω. The volume form is bi-invariant if and only if

$$dL_{g}\circ dR_{g^{-1}}\left(\omega_{e}\right)=\omega_{e},$$

or equivalently $\det\left(dL_{g}\circ dR_{g^{-1}}\right)=\det\left(Ad_{g}\right)=1$. Since SO(n) is compact, it admits a bi-invariant measure. Hence $\det(Ad_{R})=1$, and we have

$$\det\left(Ad_{\left(R,t\right)}\right)=\det\left(Ad_{R}\right)\cdot \det\left(R\right)=1.$$

We note $\mu_{G}$ the bi-invariant measure associated with ω. The fact that SE(n) is unimodular has a significant impact on the definition of statistical tools: it is possible to manipulate densities of probability distributions with respect to a canonical measure.

A convenient way to represent elements of SE(n) is to identify the isometry (R,t) with the matrix

$$\left(\begin{array}{cc} R & t\\ 0 & 1 \end{array}\right)\in GL_{n+1}\left(R\right).$$

It is easy to check that the composition of isometries corresponds to the matrix multiplication. SE(n) is thus seen as a Lie subgroup of $GL_{n+1}\left(R\right)$. Our density modelling framework is intrinsic and does not depend on a specific choice of coordinates. However, it is useful for some computations to set a reference basis. The tangent space at the identity element, noted $T_{e}SE\left(n\right)$, is spanned by the matrices of the form

$$A_{i,j}=\left(\begin{array}{cc} E_{i,j}-E_{j,i} & 0\\ 0 & 0 \end{array}\right)\;\;\mathrm{and}\;\;T_{i}=\left(\begin{array}{cc} 0 & e_{i}\\ 0 & 0 \end{array}\right)$$

where $E_{i,j}$ is the n×n matrix with a 1 at index (i,j) and zeros elsewhere and $e_{i}$ is the *i*-th basis vector of $R^{n}$. Let $B_{e}=\left(A_{i,j}\right)⋃\left(T_{i}\right)$ be the reference basis of $T_{e}SE\left(n\right)$. $B_{e}$ can be translated by left multiplication to make a left-invariant field of basis B. Depending on context *A* will denote an n×n skew-symmetric matrix or its embedding in the Lie algebra of $GL_{n+1}$, and tangent vectors will be noted with the letter *u*: u=(A,T).

Recall that a skew-symmetric matrix can be block-diagonalized with 2 by 2 rotations on the diagonal, followed by a 0 when the dimension is odd. For each *n* by *n* skew-symmetric matrix *A*, we note $\theta_{1},\ldots ,\theta_{\left\lfloor \frac{n}{2}\right\rfloor }$ the set of angles of the 2 by 2 rotations.

The identification of SE(n) with a Lie subgroup of $GL_{n+1}\left(R\right)$ makes the computation of the exponential map easy: the group exponential is simply the matrix exponential. Let *U* be the subset of $T_{e}SE\left(n\right)$ defined by

$$U=\{u=\left(A,T\right)|\;\forall i,\theta_{i}\in [-\pi ,\pi ]\}.$$

It can be checked that the exponential map on *U* is a bijection. Therefore, we can define the logarithm on SE(n) as the inverse of the exponential on *U*.

## 3. Bi-Invariant Local Linearizations

Moments and densities are defined using local linearizations of the group. Hence, to obtain bi-invariant statistics, the linearization must be compatible with left and right multiplications. This section describes why the exponential map provides such linearizations from arbitrary elements.

Though we do not use this formalism, the construction of the exponential at *g* can be viewed in the general setting of Cartan connections on Lie groups. The exponential at *g* is then the exponential of a bi-invariant connection, see [21,22,23].

### 3.1. The Exponential at Point *G*

**Figure 1:**
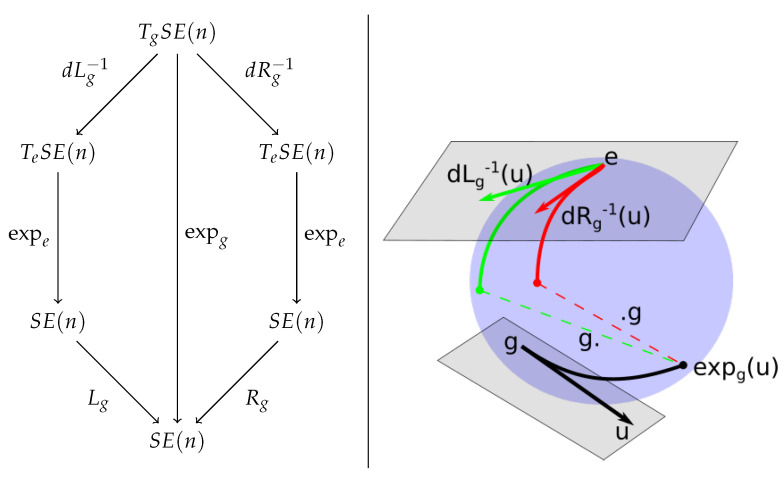
Commutation of the Adjoint/conjugation and the exponential.

Since the exponential maps the lines of the tangent space at *e* to the one parameter subgroups of SE(n), it is a natural candidate to linearize the group near the identity. To linearize the group around an arbitrary element *g*, it is possible to move *g* to the identity by a multiplication by $g^{-1}$, use the linearization at identity to obtain a tangent vector in $T_{e}SE\left(n\right)$, and map the resulting tangent vector to $T_{g}SE\left(n\right)$ with a multiplication by *g*. Fortunately, we can check that this procedure does not depend on a choice of left or right multiplication. Recall that on a Lie group,

$$g\exp\left(u\right)g^{-1}=\exp\left(Ad_{g}\left(u\right)\right)=\exp\left(dL_{g}\left(dR_{g^{-1}}\left(u\right)\right)\right)=\exp\left(dR_{g^{-1}}\left(dL_{g}\left(u\right)\right)\right),$$

where $dL_{g}$ and $dR_{g}$ are the differentials of the left and right multiplication. This property enables the transport of the exponential application to any element of the group without ambiguity on the choice of left or right multiplication,
$\exp_{g}:$$T_{g}SE\left(n\right)\to SE\left(n\right)$
$u\mapsto \exp_{g}\left(u\right)=g\times \exp\left(dL_{g^{-1}}u\right)=\exp\left(dR_{g^{-1}}u\right)g$,
see Figure 1 for a visual illustration.

Note $U_{g}\subset T_{g}SE\left(n\right)=dL_{g}\left(U\right)$ the injectivity domain of $\exp_{g}$. The logarithm $\log_{g}:SE\left(n\right)\to U_{g}$ becomes

(2) $$\begin{array}{lll} \log_{g_{0}}\left(g\right) & = & dL_{g_{0}}\left(\log\left(g_{0}^{-1}g\right)\right) \end{array}$$

(3) $$\begin{array}{cc} = & dR_{g_{0}}\left(\log\left(gg_{0}^{-1}\right)\right). \end{array}$$

**Figure 2:**
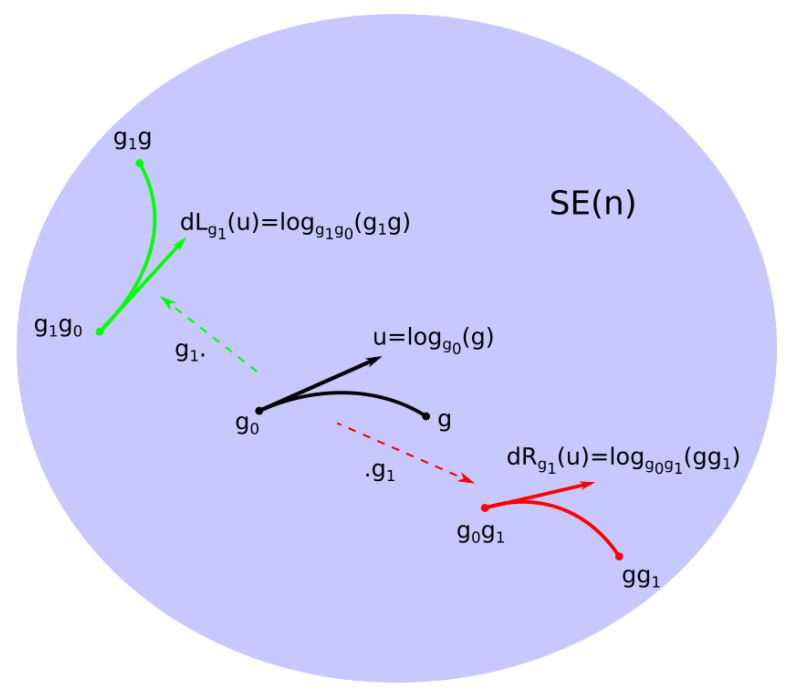
Bi-invariant linearization.

We now have a linearization of the group around an arbitrary g∈SE(n). The bi-invariant nature of the linearization is summarized in Figure 2. Independence from the choice of left or right multiplication in the definition of the exponential at an arbitrary point was the key ingredient of the definition of the bi-invariant mean in [2]. It is again a key property in our statistical model.

The strength of the exponential map is that it turns some general algebra problems into linear algebra. Once the space has been lifted to a tangent space, the problem of left and right invariances is reduced to the study of the commutation with the differentials of left and right multiplications. Since the tangent spaces do not have a canonical basis or scalar product, the manipulations we perform such as computing a mean, a covariance or estimating a density should not depend on the choice of a particular coordinate system. Hence if these manipulations commute with all the linear invertible transformations, in particular with the left and right differentials, they induce bi-invariant operations.

### 3.2. Jacobian Determinant of the Exponential

**Figure 3:**
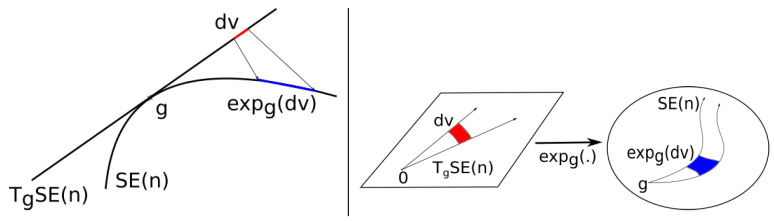
To push a density from a tangent space to the group, it is necessary to know the ratios between red and blue areas.

A measure μ on $T_{g}SE\left(n\right)$ can be pushed forward to the group using the exponential at *g*. This push-forward measure is noted $\exp_{g*}\left(\mu \right)$. Since $\exp_{g}$ commutes with the right and left actions, so does the push-forward of measures. To obtain expressions of the densities on the group, it is necessary to compute the Jacobian determinant of the exponential, see Figure 3.

Assume μ has a density *f* with respect to a Lebesgue measure of $T_{g}SE\left(n\right)$ and that its support is contained in an injectivity domain $U_{g}$ of $\exp_{g}$. The density $f_{SE(n)}$ of the measure pushed on the group is given by

$$f_{SE(n)}\left(\exp_{g}u\right)=\frac{d\exp_{g*}\left(\mu \right)}{d\mu_{G}}\left(\exp_{g}\left(u\right)\right)=\frac{1}{\left|\det(d{\left(\exp_{g}\right)}_{u})\right|}f\left(u\right)$$

where $d{\left(\exp_{g}\right)}_{u}$ is the differential of $\exp_{g}$ at the vector *u* expressed in the left-invariant reference field of basis. Since SE(n) is unimodular, i.e., $\mu_{G}$ is bi-invariant, the density of the pushed forward measure also commutes with the left and right translations of SE(n).

We now compute this Jacobian determinant at the identity element. For the sake of notation, we drop the index *e* and let $d\exp_{u}$ be the differential of the group exponential at the tangent vector *u* expressed in the bases $B_{e}$ and $B_{\exp(u)}$. $d\exp_{u}$ has the following expression (see [20,24]):

$$d\exp_{u}=dL_{\exp_{u}}\circ \left(\sum_{k\geq 0}\frac{{\left(-1\right)}^{k}}{(k+1)!}ad_{u}^{k}\right).$$

Since $\det(dL_{\exp_{u}})=1$, the Jacobian determinant of the exponential is given by the determinant of the series. Fortunately, the adjoint action can be diagonalized and the determinant can be computed explicitly. Recall that $ad_{u=(A,T)}=d{\left(Ad_{\left(R,t\right)}\right)}_{(R,t)=e}\left(A,T\right)$. Using Equation(1) we have that the matrix of $ad_{u}$ has the following form

(4) $$ad_{u}:\left(\begin{array}{cc} ad_{A} & 0\\ D & A \end{array}\right),$$

where on the left side *A* is an n×n skew-symmetric matrix, $ad_{A}$ is the adjoint map in the Lie algebra of skew-symmetric matrices, and *D* is an $\frac{n(n-1)}{2}$ by *n* matrix. Since the matrix of $ad_{\left(A,T\right)}$ is block triangular,

$$\det\left(d\exp_{u}\right)=\det\left(\sum_{k\geq 0}\frac{{\left(-1\right)}^{k}}{(k+1)!}ad_{A}^{k}\right).\det\left(\sum_{k\geq 0}\frac{{\left(-1\right)}^{k}}{(k+1)!}A^{k}\right).$$

Both determinants are obtained by diagonalizing *A* and $ad_{A}$. Take a d×d real skew-symmetric matrix *M*. There is an unitary matrix *P* such that $M=PD{\bar{P}}^{t}$, where *D* is diagonal with eigenvalues $i\lambda_{1},-i\lambda_{1},\ldots ,i\lambda_{\left\lfloor \frac{d}{2}\right\rfloor },-i\lambda_{\left\lfloor \frac{d}{2}\right\rfloor }$ with $\lambda_{i}\in R$ followed by a 0 when *d* is odd. For λ≠0 we have,

$$\sum_{k\geq 0}\frac{{\left(-1\right)}^{k}}{(k+1)!}\lambda^{k}=\frac{1-e^{-\lambda }}{\lambda },$$

and when λ=0, the left term equals 1 and the right term can be extended by continuity. The right terms are the eigenvalues of the series in *M*. Hence using the fact that the determinant of a diagonalizable matrix is the product of its eigenvalues we have

(5) $$\begin{array}{lll} \det\left(\sum_{k\geq 0}\frac{{\left(-1\right)}^{k}}{(k+1)!}M^{k}\right) & = & \prod_{i}\frac{1-e^{-i\lambda_{i}}}{i\lambda_{i}}\frac{1-e^{i\lambda_{i}}}{-i\lambda_{i}}\\ & = & \prod_{i}2\frac{1-cos(\lambda_{i})}{\lambda_{i}^{2}}. \end{array}$$

*A* is by definition skew-symmetric. Since the adjoint representation of SO(n) is compact, there is a basis of matrices such that $ad_{A}$ are skew-symmetric. Hence, Equation (5) enables the computation of $\det(d\exp_{u})$ from the eigenvalues of *A* and $ad_{A}$. The eigenvalues of $ad_{A}$ are usually obtained by computing the roots of the complexified Lie algebra of the group SO(n), see ([25], [chap. 3, sec. 8]). We provide a direct computation in Appendix A. If *n* is even, we have then

(6) $$\det\left(d\exp_{u}\right)=\prod_{i}2\frac{1-cos(\theta_{i})}{\theta_{i}^{2}}\cdot \prod_{i<j}\left(4\cdot \frac{1+cos(\theta_{i}+\theta_{j})}{{\left(\theta_{i}+\theta_{j}\right)}^{2}}\cdot \frac{1+cos(\theta_{i}-\theta_{j})}{{\left(\theta_{i}-\theta_{j}\right)}^{2}}\right)$$

and for *n* odd,

(7) $$\det\left(d\exp_{u}\right)={\left(\prod_{i}2\frac{1-cos(\theta_{i})}{\theta_{i}^{2}}\right)}^{2}\cdot \prod_{i<j}\left(4\cdot \frac{1+cos(\theta_{i}+\theta_{j})}{{\left(\theta_{i}+\theta_{j}\right)}^{2}}\cdot \frac{1+cos(\theta_{i}-\theta_{j})}{{\left(\theta_{i}-\theta_{j}\right)}^{2}}\right).$$

Let $J_{g}\left(u\right)=\left|\det\left(d\exp_{g,u}\right)\right|$. Since $\exp_{g}\left(u\right)=g\cdot \exp_{e}\left(dL_{g^{-1}}u\right)$,

$$d\exp_{g,u}=dL_{g}\circ d\exp_{e,dL_{g^{-1}}\left(u\right)}\circ dL_{g^{-1}}.$$

Furthermore,

$$dL_{g^{-1}}\left(B_{g}\right)=B_{e}\;\mathrm{and}\;B_{\exp_{g}\left(u\right)}=dL_{g}\left(B_{\exp_{e}\left(dL_{g^{-1}}\left(u\right)\right)}\right).$$

Hence expressed in the basis $B_{g}$ and $B_{\exp_{g}\left(u\right)}$, the determinant of $d\exp_{g,u}$ is given by

$$J_{g}\left(u\right)=J_{e}\left(dL_{g^{-1}}\left(u\right)\right).$$

When all tangent vectors are expressed in the left-invariant basis, it is possible to drop the subscripts and write

(8) $$J\left(A,T\right)=J\left(\theta_{1},\ldots ,\theta_{\left\lfloor \frac{n}{2}\right\rfloor },T\right)=\left|\det(d\exp_{\left(A,T\right)})\right|.$$

On SE(2) we simply have

(9) $$J\left(\theta ,T\right)=\left|2\frac{1-cos(\theta )}{\theta^{2}}\right|.$$

## 4. First and Second Moments of a Distribution on a Lie Group

### 4.1. Bi-Invariant Means

Bi-invariant means on Lie groups have been introduced by Pennec and Arsigny, see [2]. An element $\bar{g}$ in a Lie group *G* is said to be a bi-invariant mean of $g_{1},\cdots ,g_{k}\in G$ or of probability distribution μ on *G*, if

$$\sum_{i}\log_{\bar{g}}\left(g_{i}\right)=0\;\mathrm{or}\;\int_{G}\log_{\bar{g}}\left(g\right)d\mu \left(g\right)=0.$$

Observe that $\bar{g}$ is not necessarily unique, see [2,26,27] for more details. Using Equation (2), it is straightforward to check that the mean is compatible with left and right multiplications:

$$dL_{g^{\prime }}\left(\sum_{i}\log_{g}\left(g_{i}\right)\right)=\sum_{i}\log_{g^{\prime }g}\left(g^{\prime }g_{i}\right)\;\;\mathrm{and}\;\;dR_{g^{\prime }}\left(\sum_{i}\log_{g}\left(g_{i}\right)\right)=\sum_{i}\log_{gg^{\prime }}\left(g_{i}g^{\prime }\right),$$

Hence if $\sum_{i}\log_{\bar{g}}\left(g_{i}\right)=0$ we also have $\sum_{i}\log_{g^{\prime }\bar{g}}\left(g^{\prime }g_{i}\right)=0$ and $\sum_{i}\log_{\bar{g}g^{\prime }}\left(g_{i}g^{\prime }\right)=0$.

### 4.2. Covariance in a Vector Space

In this section, the bold letter *u* represents a vector and the letter *u* its coordinate in a basis.

Let us recall the definition of the covariance of a distribution on a vector space in a coordinate system. Let $e_{1},\ldots ,e_{n}$ be a basis of the vector space *V* and μ a distribution on *V*. The covariance of μ in *V* is defined by

$$\Sigma =E_{\mu }\left(\left(u-\bar{\mu }\right){\left(u-\bar{\mu }\right)}^{t}\right)=\int_{V}\left(u-\bar{\mu }\right){\left(u-\bar{\mu }\right)}^{t}d\mu \left(u\right),$$

where *u* and $\bar{\mu }$ are the coordinate expressions of the vector *u* and the average of μ and $E_{\mu }\left(\right)$ is the expectation with respect to μ.

Let $K:R_{+}\to R_{+}$ be such that K(∥x∥) is a probability density on $R^{n}$ whose covariance matrix in the canonical basis is the identity matrix, and μ be the distribution on *V* whose density is

(10) $$\frac{d\mu }{d\lambda_{e}}\left(u\right)=\frac{1}{\det\left(\sqrt{\Sigma }\right)}K\left(\sqrt{u^{t}\Sigma^{-1}u}\right).$$

where $\lambda_{e}$ is the Lebesgue measure induced by $e_{1},\ldots ,e_{n}$. It is easy to check that the covariance of μ is Σ.

Since the tangent space of a Lie group does not have a canonical basis, it is sometimes useful to define objects independently of coordinates. The coordinate free definition of the covariance becomes

$$\Sigma =\int_{V}\left(u-\bar{\mu }\right)⊗\left(u-\bar{\mu }\right)d\mu \left(u\right).$$

Recall that V⊗V is naturally identified with the space of bilinear forms on $V^{*}$. Let $B^{*}$ be the bilinear form on $V^{*}$ associated with Σ. If $B^{*}$ is positive definite, it induces an isomorphism between $V^{*}$ and *V* and is then naturally identified with a bilinear form *B* on *V*. The definition μ in Equation (10) becomes

$$\frac{d\mu }{d\lambda_{B}}\left(u\right)=K\left(\sqrt{B\left(u,u\right)}\right),$$

where $\lambda_{B}$ is the Lebesgue measure on *V* induced by *B*. In this formulation it clearly appears that μ does not depends on a basis.

### 4.3. Covariance of a Distribution on SE(N)

**Figure 4:**
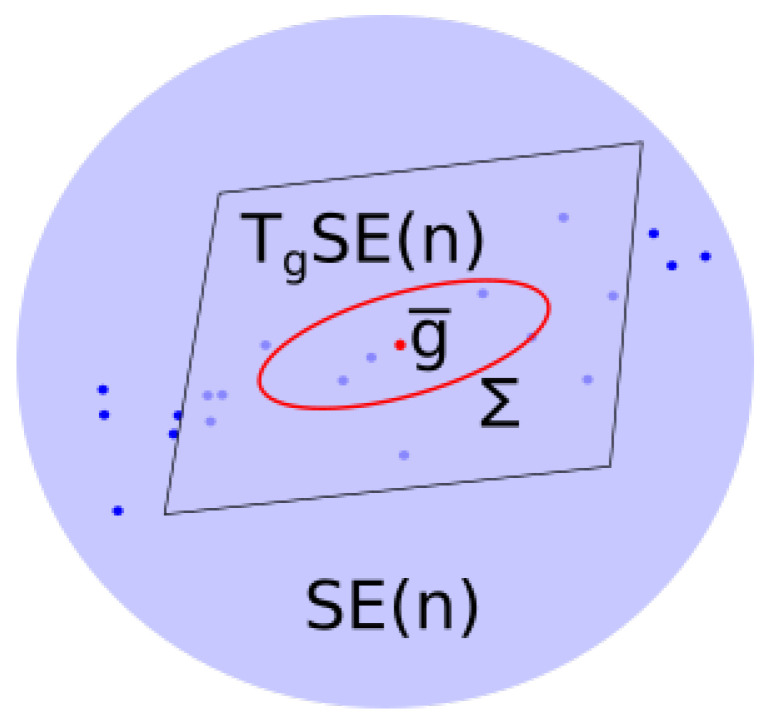
Covariance of an empirical measure.

Let μ be a distribution on SE(n) such that its bi-invariant mean $\bar{g}$ is uniquely defined. The covariance tensor of μ is defined as

$$\Sigma =E_{\mu }\left(\log_{\bar{g}}\left(g\right)⊗\log_{\bar{g}}\left(g\right)\right)=\int_{SE(n)}\log_{\bar{g}}\left(g\right)⊗\log_{\bar{g}}\left(g\right)d\mu \left(g\right)\in T_{\bar{g}}SE\left(n\right)⊗T_{\bar{g}}SE\left(n\right),$$

see Figure 4 for a visual illustration.

Again, using Equation (2) and the bi-invariance of the mean, the compatibility of the covariance with left and right multiplication is straightforward. Note g·Σ and Σ·g the pushforwards by left and right multiplication by *g* of the tensor Σ. We have then

$$\begin{array}{lll} g^{\prime }\cdot \Sigma & = & E_{\mu }\left(dL_{g^{\prime }}\left(\log_{\bar{g}}\left(g\right)\right)⊗dL_{g^{\prime }}\left(\log_{\bar{g}}\left(g\right)\right)\right)\\ & = & E_{\mu }\left(\log_{g^{\prime }\bar{g}}\left(g^{\prime }g\right)⊗\log_{g^{\prime }\bar{g}}\left(g^{\prime }g\right)\right)\\ & = & \Sigma^{\prime } \end{array}$$

where $\Sigma^{\prime }$ is the covariance of the distribution $g^{\prime }\cdot \mu$, the push-forward of μ by $L_{g^{\prime }}$. The same goes for right multiplications. However, it is important to note that for a covariance Σ defined on $T_{g}SE\left(n\right)$, pushing the covariance to the tangent space at identity using left and right multiplication usually gives different results:

$$g^{-1}\cdot \Sigma =Ad_{g^{-1}}\left(\Sigma \cdot g^{-1}\right)\neq \Sigma \cdot g^{-1},$$

where $Ad_{g}\left(\cdot \right)$ is interpreted as the map on tensors induced by the adjoint representation.

For two distributions $\mu_{1}$ and $\mu_{2}$ with different means, their covariance tensors are objects defined in different tangent spaces. The collection of all these spaces form the tangent bundle TSE(n), and covariances are identified to points in the tensor bundle TSE(n)⊗TSE(n).

In the reference field of basis B, the covariance Σ has a matrix Σ given by

$$\Sigma =\int_{SE(n)}\log_{\bar{g}}\left(g\right)\log_{\bar{g}}{\left(g\right)}^{t}d\mu \left(g\right).$$

In principal geodesic analysis, the matrix Σ is sometimes referred to as a linearized quantity in contrast to the *exact* principal geodesic analysis, see [28].

## 5. Statistical Models for Bi-Invariant Statistics

### 5.1. The Model

Let $K:R_{+}\to R_{+}$ be such that

(i) $\int_{R^{n}}K\left(∥u∥\right)du=1$
(ii) $\int_{R^{n}}uu^{t}K\left(∥u∥\right)du=I,\;I\;\mathrm{being}\;\mathrm{the}\;n\times n\;\mathrm{identity}\;\mathrm{matrix}$
(iii) K(x>a)=0forsomea∈R.

Condition (i) imposes that K(∥u∥) is a probability density on $R^{n}$, condition (ii) that the covariance matrix is the identity matrix and condition (iii) that it has a bounded support.

**Figure 5:**
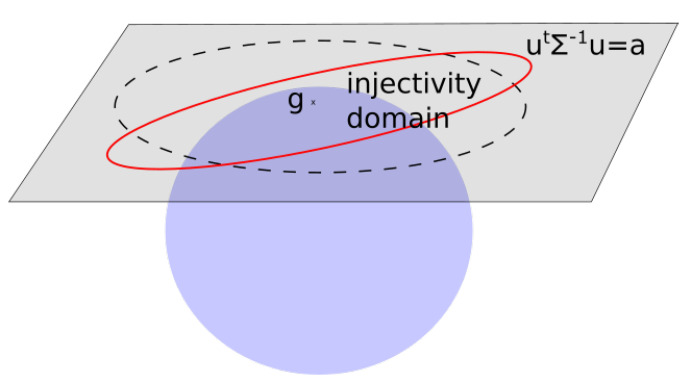
$\Sigma \notin C_{g}$.

The statistical model is defined by pushing densities of the form K(∥u∥) from tangent spaces to the group via the exponential, where the Euclidean norms on tangent spaces are parameters of the distributions. To avoid summing densities over the multiple inverse images of the exponential map, it is convenient to deal with densities K(∥u∥) whose support are included in injectivity domains, hence the (iii) requirement. Let $C_{g}$ be the set of covariance matrices compatible with the injectivity domain $U_{g}$,

$$C_{g}=\left\{\Sigma |\forall u\notin U_{g},u^{t}\Sigma^{-1}u>a\right\},$$

see Figure 5. When covariance matrices are expressed in the left-invariant reference basis, the set $C_{g}$ is the same for all *g* and the subscript can be dropped.

When $\Sigma \in C_{g}$, the support of the probability distribution μ on $T_{g}SE\left(n\right)$ defined by

$$\frac{d\tilde{\mu }}{d\lambda_{g}}\left(u\right)=\frac{1}{\sqrt{\det(\Sigma )}}K\left(\sqrt{u^{t}\Sigma^{-1}u}\right),$$

where $\tilde{\mu }$ is the lift of μ by $\log_{g}$, is contained in $U_{g}$. Here $\lambda_{g}$ denotes the Lebesgue measure of $T_{g}SE\left(n\right)$. The density of the push-forward of μ is then

(11) $$f\left(\exp_{g}\left(u\right)\right)=\frac{1}{J\left(u\right)\sqrt{\det(\Sigma )}}K\left(\sqrt{u^{t}\Sigma^{-1}u}\right),$$

or, expressed at $g^{\prime }\in SE\left(n\right)$,

(12) $$f\left(g^{\prime }\right)=\frac{1}{J\left(\log_{g}\left(g^{\prime }\right)\right)\sqrt{\det(\Sigma )}}K\left(\sqrt{\log_{g}{\left(g^{\prime }\right)}^{t}\Sigma^{-1}\log_{g}\left(g^{\prime }\right)}\right),$$

where *J* is given in Equation (8). The set of such probability densities when *g* and Σ vary form a natural parametric statistical model:

$$M=\left\{f_{g,\Sigma }:g\in SE\left(n\right)\;\mathrm{and}\;\Sigma \in C_{g}\right\}.$$

The commutation relations of Section 3.1 imply that M is closed under left and right multiplications. The fact that *g* and Σ are the moments of $f_{g,\Sigma }$ plays a major role in the relevance of the model M. This fact holds when Σ is small enough, a more precise result should follow in a future work.

### 5.2. Sampling Distributions of M

An advantage of constructing distributions from tangent spaces is that they are easy to sample: it suffices to be able to sample from the probability density *p* on R proportional to K(x), p∝K. Recall that the dimension of tangent spaces is $d=\frac{n(n+1)}{2}$. Let $v_{i}={\left(x_{1,i},\ldots ,x_{d,i}\right)}^{t}$ be random column vectors with $x_{k,l}$ i.i.d. reals distributed according to *p*. Then the vectors

$$u_{i}=\Sigma^{\frac{1}{2}}v_{i}$$

are i.i.d. of density $\frac{1}{\sqrt{\det(\Sigma )}}K\left(\sqrt{u^{t}\Sigma^{-1}u}\right)$ on $R^{d}$, and the points

$$g_{i}=\exp_{g}\left(u_{i}\right)$$

are i.i.d. according to the density $f_{g,\Sigma }$ on SE(n).

### 5.3. Evaluation of the Convergence of the Moment-Matching Estimator

All the experiments in this section were performed using the Python package geomstats, see [29], available at http:geomstats.ai. Let $g_{1},\ldots ,g_{k}$ be points in SE(n) with a unique bi-invariant mean $\hat{g}$ and such that the empirical covariance

$$\hat{\Sigma }\left(g_{1},\ldots ,g_{k}\right)=\frac{1}{k}\sum_{i}\log_{\hat{g}}\left(g_{i}\right)\log_{\hat{g}}{\left(g_{i}\right)}^{t}$$

is contained in $C_{\hat{g}}$ and that the moments of $f_{\hat{g},\hat{\Sigma }}\mu_{G}$ are $\left(\hat{g},\hat{\Sigma }\right)$. The compatibilities with left multiplications,

$$\;f_{g^{\prime }g,g^{\prime }\Sigma }=g^{\prime }\cdot f_{g,\Sigma }\;\;\mathrm{and}\;\hat{\Sigma }\left(g^{\prime }\cdot g_{1},\ldots ,g^{\prime }\cdot g_{k}\right)=g^{\prime }.\hat{\Sigma }\left(g_{1},\ldots ,g_{k}\right),$$

and right multiplications, implies that the maximum likelihood and the moment-matching estimators are bi-invariant.

On the one hand, finding the maximum likelihood estimation when $g_{1},\ldots ,g_{k}$ are i.i.d. requires an optimization procedure. On the other hand, matching moments is straightforward, provided that the moments of $f_{\bar{g},\Sigma }$ are $\left(\bar{g},\Sigma \right)$. In most cases, this moment-matching estimator is expected to have reasonable convergence properties; however there are currently no theoretical results on the convergence of bi-invariant means and covariance on Lie groups. Hence for now it is only possible to provide empirical convergence on specific examples. Let

(13) $$K\left(x\right)=\frac{3}{4\pi 5^{3/2}}1_{\left[0,\sqrt{5}\right]}\left(x\right),\;\Sigma_{1}=\left(\begin{array}{ccc} 1 & 0 & 0\\ 0 & 1 & 0\\ 0 & 0 & 1 \end{array}\right),\;\Sigma_{2}=\left(\begin{array}{ccc} 0 & -1 & 0\\ 1 & 0 & 0\\ 0 & 0 & 1 \end{array}\right)\left(\begin{array}{ccc} 0.5 & 0 & 0\\ 0 & 0.2 & 0\\ 0 & 0 & 1 \end{array}\right)\left(\begin{array}{ccc} 0 & 1 & 0\\ -1 & 0 & 0\\ 0 & 0 & 1 \end{array}\right).$$

The function *K* verifies (i), (ii) and (iii) of Section 5.1. Since $\sqrt{5}<\frac{\pi }{2}$, $\Sigma_{1}$ and $\Sigma_{2}$ are admissible covariances, $\Sigma_{1,2}\in C$. $\Sigma_{2}$ is chosen such that it correlates the rotation and translation coordinates.

Given a set of i.i.d. samples $g_{1},\ldots ,g_{k}$ of the density $f_{e,\Sigma }$, the estimated density of the moment-matching estimator is $f_{\hat{g},\hat{\Sigma }}$. For the sake of notations, we drop subscripts and simply write *f* and $\hat{f}$. To characterize the convergence of the estimator, we compare the convergence of $\hat{f}$ on SE(2) with the analogous moment-matching estimator on $T_{e}SE\left(2\right)∼R^{3}$ using the samples $\log\left(g_{1}\right),\ldots ,\log\left(g_{k}\right)$.

**Figure 6:**
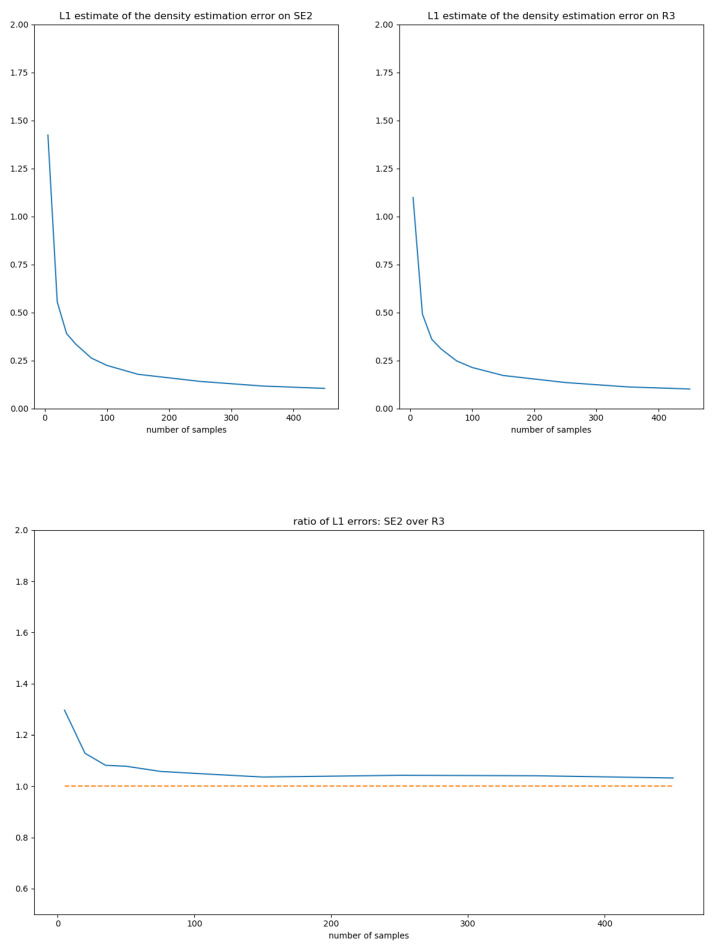
$L^{1}$ errors and their ratios on SE(2) and $T_{e}SE\left(2\right)∼R^{3}$ for the covariance $\Sigma_{1}$, see Equation (13).

**Figure 7:**
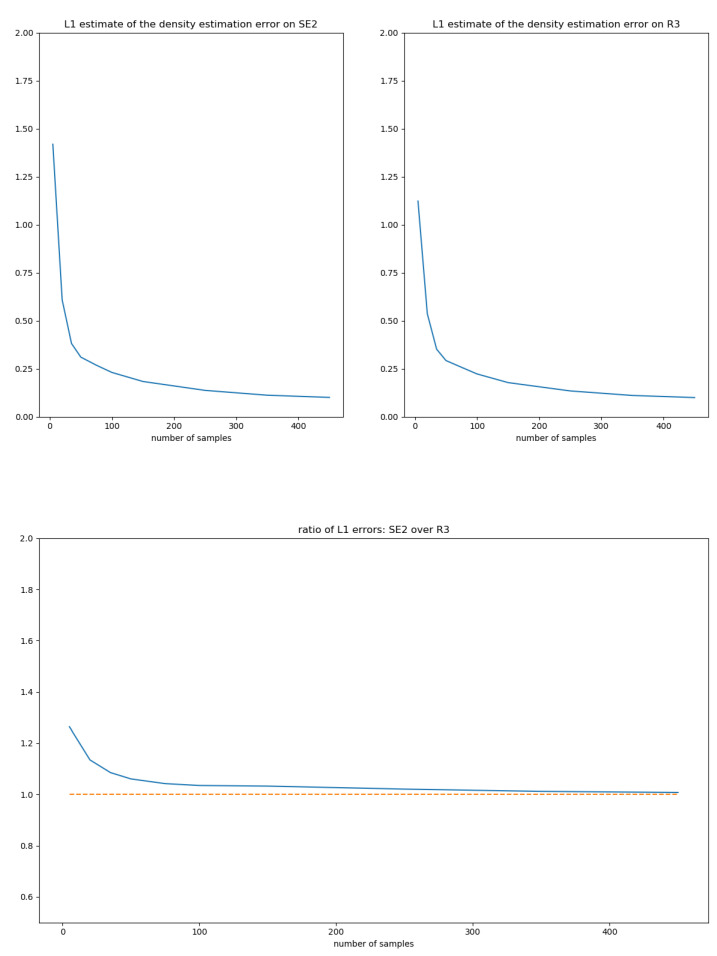
$L^{1}$ errors and their ratios on SE(2) and $T_{e}SE\left(2\right)∼R^{3}$ for the covariance $\Sigma_{2}$, see Equation (13).

Any $L^{p}$ distance between densities provides a way to evaluate the convergence in a bi-invariant way. The $L^{1}$ is particularly meaningful in the context of probabilities and presents the advantage of being independent from a reference measure. Therefore, we evaluated the expectation of the $L^{1}$ distance to *f*:

$$e_{k}=E_{f}\left(\int_{SE(2)}\left|f\left(g\right)-\hat{f}\left(g\right)\right|d\mu_{G}\left(g\right)\right),$$

and the Euclidean analogous, where *k* is the number of samples of *f*. The integrals over SE(2) can be estimated using a Monte-Carlo sampling adapted to the distributions. Indeed,

(14) $$\begin{array}{lll} \int \left|f-\hat{f}\right|d\mu_{G} & = & \int_{S}\left|1-\frac{\hat{f}}{f}\right|fd\mu_{G}+\int_{S^{c}}\hat{f}d\mu_{G} \end{array}$$

(15) $$\begin{array}{cc} = & E_{f}\left(\left|1-\frac{\hat{f}}{f}\right|\right)+1-E_{f}\left(\frac{\hat{f}}{f}\right) \end{array}$$

(16) $$\begin{array}{cc} \approx & 1+\sum_{i}\left(\left|1-\frac{\hat{f}}{f}\left(u_{i}\right)\right|-\frac{\hat{f}}{f}\left(u_{i}\right)\right) \end{array}$$

where *S* is the support of *f* and the $\left(u_{i}\right)$ are i.i.d. samples of *f*. The $L^{1}$ distances between *f* and $\hat{f}$ are estimated using 5000 Monte-Carlo samples, and the expectation of the $L^{1}$ distance is estimated using 200 estimates $\hat{f}$. Figure 6 depicts the decay of the expected $L^{1}$ distance with the number of samples for the SE(2) and $R^{3}$ cases using the covariance $\Sigma_{1}$ and Figure 7 using the covariance $\Sigma_{2}$. For a given covariance Σ, the error decay on SE(2) and $R^{3}$ seem to be asymptotically related by a multiplicative factor close to 1. Future work should focus on gaining insights into the phenomena underlying the error decay in the general case.

## 6. Conclusion and Perspectives

In this paper, we have described a statistical model M of densities for bi-invariant statistics on SE(n). Even though we do not provide convergence rates, we showed experimentally on an example that the density estimation on SE(2) behaves similarly to the estimation on $R^{3}$. Further works will focus on a deeper analysis of the performance of the moment-matching estimator, on proposing detailed algorithms to estimate densities in a mixture model, and on generalizing the construction to other Lie groups.

## Appendix A. Eigenvalues of *ad_A_*

Let A be the set of skew-symmetric n×n matrices. Let A∈A and $ad_{A}:X\in A\mapsto AX-XA\in A$. We aim at computing the eigenvalues of $ad_{A}$. Since *A* is in A, there exists an orthogonal matrix *P* such that $D=P^{-1}AP$ is a matrix with vanishing entries outside of $\left\lfloor \frac{n}{2}\right\rfloor$ 2 by 2 matrices $A_{j}=\left(\begin{array}{cc} 0 & -a_{j}\\ a_{j} & 0 \end{array}\right)$ along the diagonal. If *n* is even, the eigenvalues of $ad_{A}$ are the numbers

$$i\left(\pm a_{j}\pm a_{k}\right),\;1\leq j<k\leq \frac{n}{2}$$

$$0,\;\mathrm{with}\;\mathrm{multiplicity}\;\frac{n}{2}.$$

If *n* is odd the eigenvalues of $ad_{A}$ are the numbers

$$i\left(\pm a_{j}\pm a_{k}\right),\;1\leq j<k\leq \frac{n-1}{2}$$

$$\pm a_{j},\;1\leq j\leq \frac{n-1}{2},$$

$$0,\;\mathrm{with}\;\mathrm{multiplicity}\;\frac{n-1}{2}.$$

**Proof.** Let $g_{P}\left(X\right)=PXP^{-1}$. Since $g_{P}$ is invertible and that $ad_{A}=g_{P}\circ ad_{D}\circ g_{P}^{-1}$, $ad_{A}$ and $ad_{D}$ have the same eigenvalues. Consider first the case *n* odd. Let X∈A, *X* can be decomposed in $\frac{n-1}{2}\times \frac{n-1}{2}$ 2 by 2 sub-matrices $B_{i,j}$, $\frac{n-1}{2}$ 1 by 2 sub-matrices $u_{j}$ on the last line of *X*, and $\frac{n-1}{2}$ 2 by 1 $u_{j}^{t}$ on the last column, and a 1 by 1 sub-matrix $x=X_{n,n}$. $Y=ad_{A}\left(X\right)$ can be decomposed the same way in sub-matrices $c_{i,j},v_{i,j},v_{i,j}^{t}$ and $y=Y_{n,n}$. With the block products we obtain,

$$C_{i,j}=A_{i}B_{i,j}-B_{i,j}A_{i},\;1\leq j\leq k\leq \frac{n-1}{2}$$

$$v_{j}=-u_{j}A_{j}\;1\leq j\leq \frac{n-1}{2},$$

y=0.

it follows that each subspace $A_{ij,i\neq j}$ with vanishing entries outside the 2 by 2 blocks ij and ji, are $ad_{D}$ stable. These spaces are four-dimensional and direct calculation shows that the eigenvalues of $ad_{D}$ restricted to these spaces are $i(\pm a_{i}\pm a_{j})$. The subspace $A_{i}$ defined by the blocks $u_{i}$ and $u_{i}^{t}$ are stable as well, and the computation shows that the corresponding eigenvalues are $i(\pm a_{j})$. $ad_{D}$ restricted to blocks $A_{i,i}$ vanishes, thus 0 is of multiplicity $\frac{n-1}{2}$. In the *n* even case, only the eigenvalues associated with blocks $A_{i,j}$ remain. □
